# Reduced liver damage and fibrosis with combined SCD Probiotics and intermittent fasting in aged rat

**DOI:** 10.1111/jcmm.18014

**Published:** 2023-10-28

**Authors:** Hikmet Taner Teker, Taha Ceylani, Seda Keskin, Gizem Samgane, Burcu Baba, Eda Acıkgoz, Rafig Gurbanov

**Affiliations:** ^1^ Department of Medical Biology and Genetics Ankara Medipol University Ankara Turkey; ^2^ Department of Molecular Biology and Genetics Muş Alparslan University Muş Turkey; ^3^ Department of Food Quality Control and Analysis Muş Alparslan University Muş Turkey; ^4^ Department of Histology and Embryology Van Yuzuncu Yil University Van Turkey; ^5^ Department of Bioengineering Bilecik Şeyh Edebali University Bilecik Turkey; ^6^ Department of Medical Biochemistry Yüksek İhtisas University Ankara Turkey; ^7^ Central Research Laboratory (BARUM) Bilecik Şeyh Edebali University Bilecik Turkey

**Keywords:** fibrosis, FTIR, intermittent fasting, liver, rat, SCD probiotics

## Abstract

This study aimed to examine the impact of SCD Probiotics supplementation on liver biomolecule content and histological changes during a 30‐day intermittent fasting (IF) program in 24‐month‐old male Sprague–Dawley rats. Rats underwent 18‐h daily fasting and received 1 × 10^8^ CFU of SCD Probiotics daily. Liver tissue biomolecules were analysed using FTIR Spectroscopy, LDA, and SVM techniques, while histopathological evaluations used Haematoxylin and eosin and Masson trichrome‐stained tissues. Blood samples were collected for biochemical analysis. Gross alterations in the quantity of biomolecules were observed with individual or combined treatments. LDA and SVM analyses demonstrated a high accuracy in differentiating control and treated groups. The combination treatments led to the most significant reduction in cholesterol ester (1740 cm^−1^) and improved protein phosphorylation (A_1239_/A_2955_ and A_1080_/A_1545_) and carbonylation (A_1740_/A_1545_). Individually, IF and SCD Probiotics were more effective in enhancing membrane dynamics (Bw_2922_/Bw_2955_). In treated groups, histological evaluations showed decreased hepatocyte degeneration, lymphocyticinfiltration, steatosis and fibrosis. Serum ALP, LDH and albumin levels significantly increased in the SCD Probiotics and combined treatment groups. This study offers valuable insights into the potential mechanisms behind the beneficial effects of IF and SCD Probiotics on liver biomolecule content, contributing to the development of personalized nutrition and health strategies.

## 1 INTRODUCTION

Intermittent fasting (IF) is a dietary pattern involving periods of food restriction and normal eating. It has gained popularity recently due to its potential health benefits, including anti‐aging and rejuvenation effects.^1^, ^2^ Studies have shown that IF can improve biomarkers of aging, such as insulin sensitivity, inflammation and oxidative stress. One of the key benefits of IF is its impact on liver function. The liver plays a vital role in regulating metabolism and removing toxins from the body. However, the liver can become damaged and less efficient over time, leading to various health problems, including liver disease, obesity and diabetes. IF has been shown to improve liver function by reducing fat accumulation, increasing insulin sensitivity and promoting the production of new liver cells.^3^, ^4^ Additionally, IF has been linked to cellular rejuvenation. During fasting, the body enters a state of autophagy, where damaged and dysfunctional cells are broken down and recycled. This process helps to remove cellular waste and improve cellular function, which can have anti‐aging effects.^4^ IF has the potential to promote healthy aging and rejuvenation by improving liver function and promoting cellular regeneration.^5^

Probiotics are living microorganisms that can offer numerous health benefits when consumed in sufficient quantities. Among their many benefits, probiotics can support healthy aging, promote rejuvenation, and aid liver health.^6^ The gut microbiome changes with aging, including a reduction in beneficial bacteria associated with the onset of age‐related diseases such as metabolic disorders, cardiovascular disease and inflammation.^7^, ^8^ Probiotics have been found to enhance gut microbiota, boost immune function, and reduce inflammation, thus decreasing the risk of such diseases.^9^, ^10^ The liver plays a vital role in metabolism, detoxification and energy storage. However, liver function typically declines with age, which can lead to liver diseases such as non‐alcoholic fatty liver disease (NAFLD). Fortunately, probiotics have been shown to impact liver health positively. Research indicates that probiotics can assist in removing toxins from the liver and prevent various liver health issues, including NAFLD.^11^ Furthermore, probiotics possess anti‐inflammatory properties that can prevent liver inflammation.^12^ In a randomized controlled trial, probiotic supplementation improved liver function and reduced the risk of liver disease in elderly participants.^13^ Given the favourable effects of probiotics on gut microbiota, immune function, liver health and skin rejuvenation, they can be an essential component of healthy aging. Therefore, incorporating probiotics into the diet may be an essential strategy for maintaining good health in old age.^14^

This study investigates the synergistic effects of IF and SCD Probiotics on histopathology and biomolecular profiles in aged rat liver tissue. By scrutinizing biomolecular structures histological changes, and utilizing sophisticated analytical methods, we aim to unveil the potential advantages of these treatments in alleviating age‐related liver deterioration. The insights obtained from this research may lay the foundation for future exploration of the therapeutic potential of IF and SCD Probiotics in addressing age‐related liver conditions and enhancing the quality of life, especially for the elderly population.

## 2 MATERIAL METHOD

This section is presented in the Supporting Information.

## 3 RESULTS

### 3.1 The body weight, water and food consumption of animals

**FIGURE 1:**
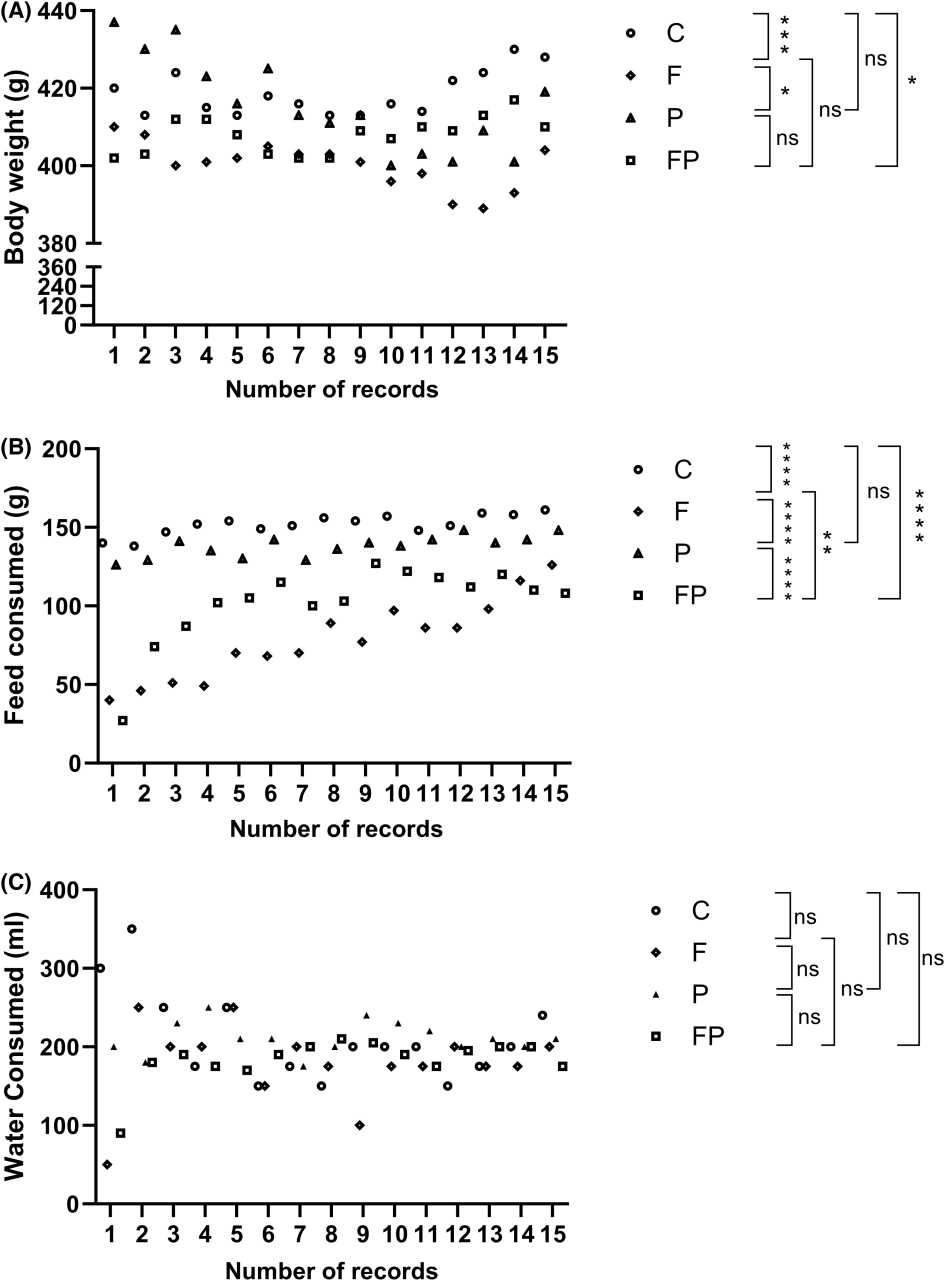
Effects of intermittent fasting, SCD probiotics supplementation, and combined treatment on: (A) Body weight: Significant difference among groups (*p* < 0.0001). Comparisons: C versus F (*p* < 0.0001), C versus P (*p* = 0.1732), C versus FP (*p* = 0.0023), F versus P (*p* < 0.0001), F versus FP (*p* = 0.0418), and P versus FP (*p* = 0.0394), (B) Food consumption: Significant difference among groups (*p* < 0.0001). Comparisons: C versus F (*p* < 0.0001), C versus P (*p* = 0.1732), C versus FP (*p* < 0.0001), F versus P (*p* < 0.0001), F versus FP (*p* = 0.00538), and P versus FP (*p* < 0.0001), (C) Water consumption: No significant difference among groups (*p* = 0.607). C (control), F (Intermittent fasting), P (SDC Probiotics), and the FP applications (in which the Intermittent fasting and SCD Probiotics were applied together).

As a result of anova performed between the groups, it was observed that there was a significant difference in terms of body weights (*p* = 0.002). When the groups are compared, C (control) versus F (fasting) (*p* = 0.0001), C versus P (SCD Probiotics) (*p* = 0.4298), C versus FP (fasting + probiotics) (*p* = 0.0236), F versus P (*p* = 0.0127), F versus FP (*p* = 0.0369), and P versus FP (*p* = 0.0369), and P versus FP (*p* = 0.4926) (Figure 1A). anova analysis also shows a significant difference in terms of feed overall consumption (*p* < 0.0001). The difference between the groups in terms of feed consumption is C versus F (*p* < 0.0001), C versus P (*p* = 0.1418), C versus FP (*p* < 0.0001), F versus P (*p* < 0.0001), F versus FP (*p* = 0.0050) and P versus FP (*p* < 0.0001) (Figure 1B). However, there was no significant difference in water consumption according to anova results (*p* = 0.1132) (Figure 1C).

### 3.2 The liver tissue lipid, protein and nucleic acid profiles showed differences in all groups

**FIGURE 2:**
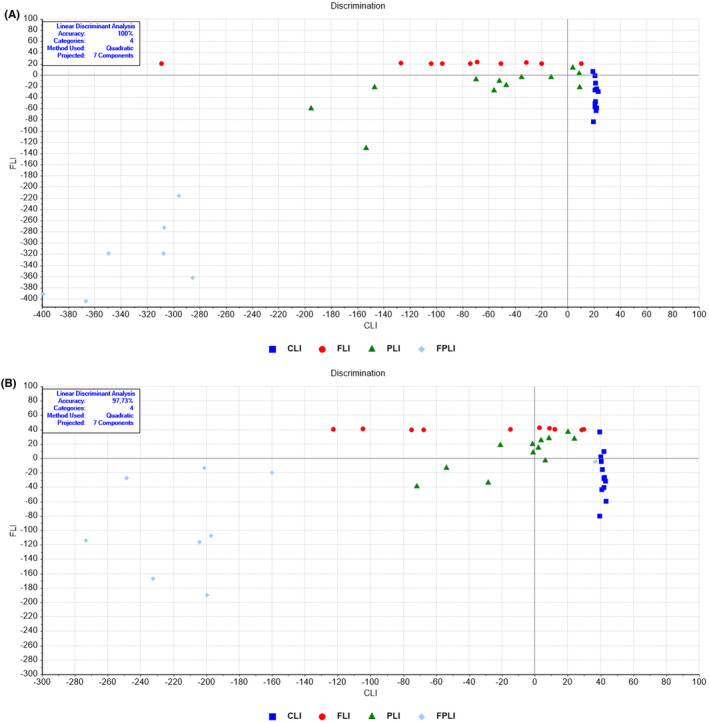
LDA discrimination plots for liver samples in the (A) full (4000–650 cm^−1^) and (B) lipid (3000–2700 cm^−1^) spectral regions. CLI (control), FLI (Intermittent Fasting), PLI (SDC Probiotics), and the FPLI applications (in which the intermittent fasting and SCD Probiotics were applied together).

The LDA method was utilized to analyse the data and found that the control group (CLI) and intermittent fasting (FLI), SDC Probiotics (PLI), and the FPLI group (in which the intermittent fasting and SCD Probiotics were applied together) were significantly different in terms of their overall biomolecule content with a 100% accuracy (Figure 2A, Tables S1 and S2). The LDA discrimination plot clearly demonstrates that the data for the control group and the groups receiving different applications are clustered in entirely different regions (Figure 2A). Similarly, significant differentiation was observed in lipid profiles, with a 97.73% accuracy rate (Figure 2B, Tables S3 and S4). The lipid data for the control and groups receiving different applications were separated (Figure 2B), with the greatest separation observed in the FPLI group. Moreover, the protein and nucleic acid content analysis showed a significant differentiation, with 100% accuracy rates (Figures S1 and S2, Tables S5–S8). SVM classification demonstrated 90.91% training and 79.54% validation accuracies for whole biomolecules (Figure S3).

**FIGURE 3:**
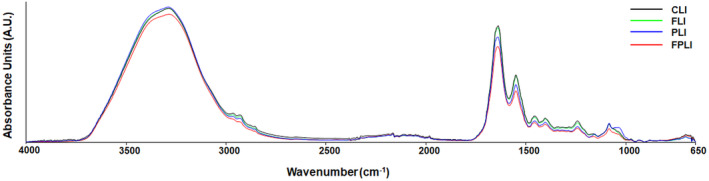
Baseline‐corrected average infrared spectra in the 4000–650 cm^−1^ spectral region for liver tissues. CLI (control), FLI (Intermittent Fasting), PLI (SDC Probiotics), and the FPLI applications (in which the intermittent fasting and SCD Probiotics were applied together).

**FIGURE 4:**
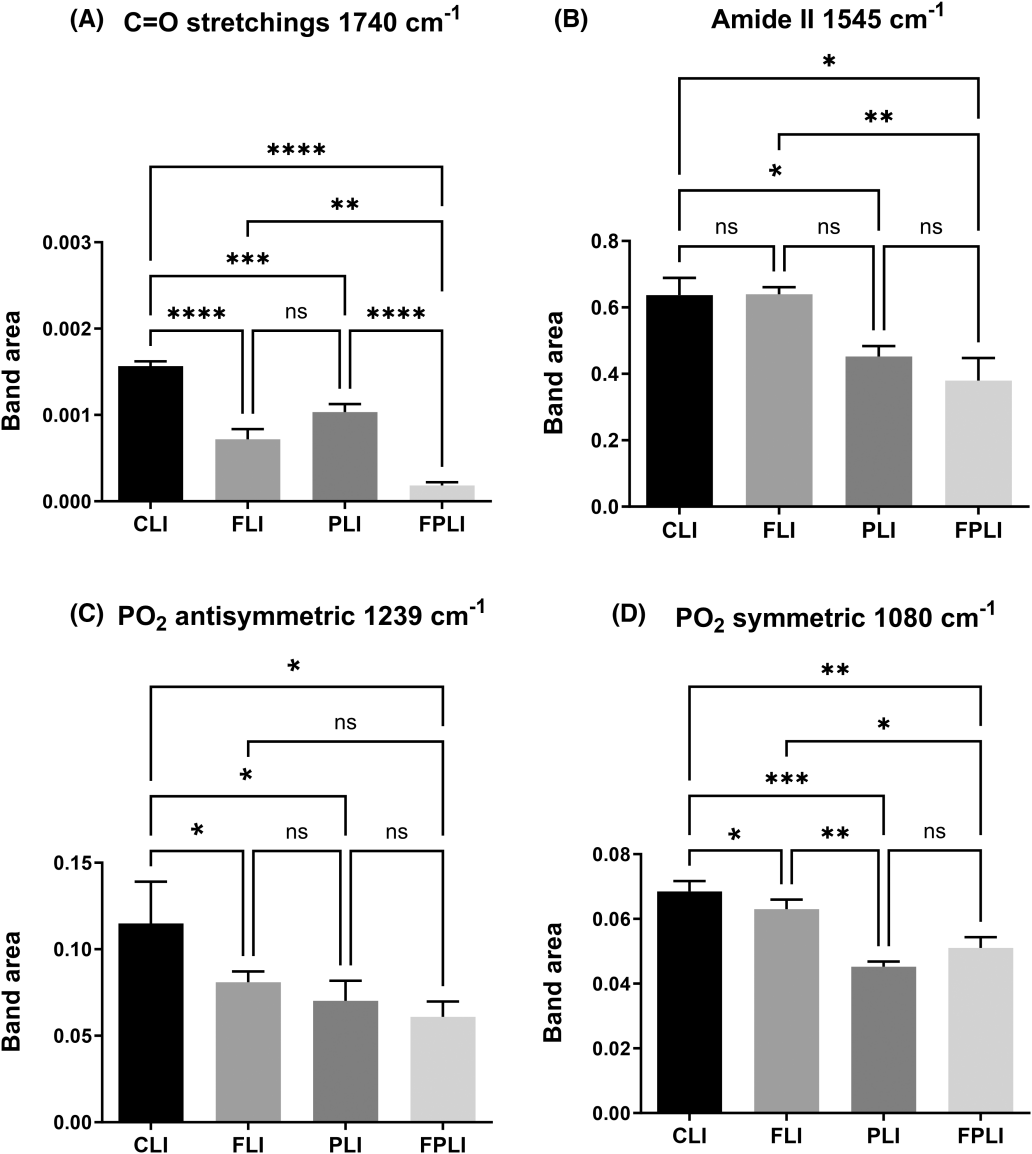
The quantitative changes in liver‐associated spectrochemical parameters. The band areas for (A) lipid carbonyl (C=O stretchings, 1740 cm^−1^), (B) Amide II (1545 cm^−1^), (C) PO_2_ antisymmetric (1239 cm^−1^), and (D) PO_2_ symmetric (1080 cm^−1^). CLI (control), FLI (Intermittent Fasting), PLI (SDC Probiotics), and the FPLI applications (in which the intermittent fasting and SCD Probiotics were applied together).

Figure 3 demonstrates the average spectra for CLI, FLI, PLI and FPLI groups over the entire infrared region (4000–650 cm^−1^), presenting various spectrochemical bands associated with specific functional groups of biomolecules. The areas of the bands at 1740 cm^−1^ (C=O stretching: cholesterol ester), 1545 cm^−1^, 1239 cm^−1^ (PO_2_ antisymmetric stretching: nucleic acids), and 1080 cm^−1^ (PO_2_ symmetric stretching: nucleic acids, phospholipid) positions^15^ changed significantly (Figure 4). It was found that the area of the band at 1740 cm^−1^ decreased considerably in all groups. IF was more effective than the use of SCD Probiotics. At the same time, the greatest reduction was seen due to the combination of IF with SCD Probiotics (Figure 4A). There was no significant change in the area of the Amide II band at 1545 cm‐1 with IF administration. Still, a considerable reduction appeared due to SCD Probiotics and a combination of IF with SCD Probiotics (Figure 4B). The area of PO_2_ bands at 1239 cm^−1^ (antisymmetric stretching) and 1080 cm^−1^ (symmetric stretching) positions was significantly reduced in all treatment groups (Figure 4C,D).

**FIGURE 5:**
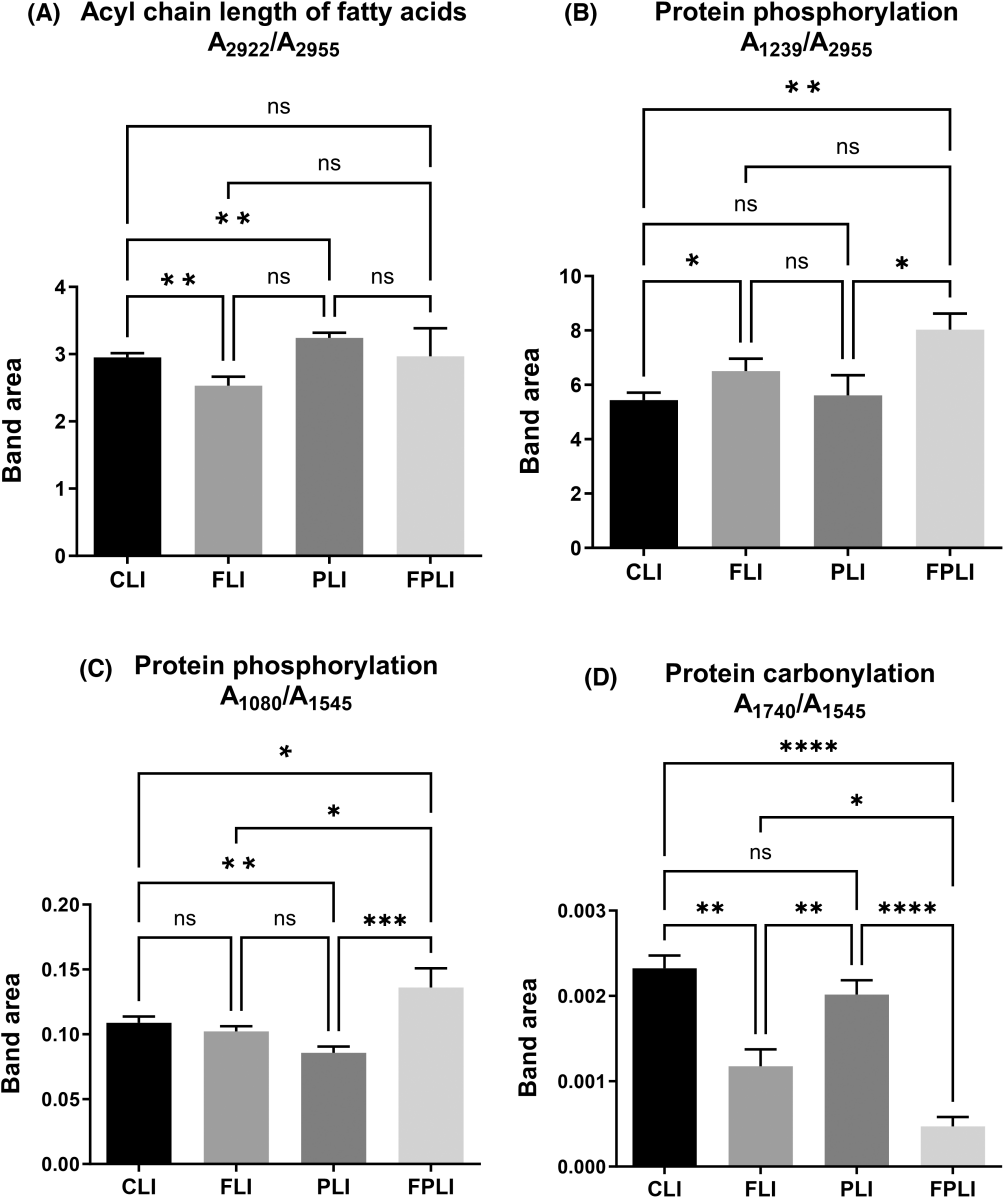
The quantitative changes in liver‐associated spectrochemical parameters. The band area ratios for (A) acyl chain length of fatty acids (A_2922_/A_2955_), (B) protein phosphorylation (A_1239_/A_2955_), (C) protein phosphorylation (A_1080_/A_1545_), and (D) protein carbonylation (A_1740_/A_1545_). CLI (control), FLI (Intermittent Fasting), PLI (SDC Probiotics), and the FPLI applications (in which the intermittent fasting and SCD Probiotics were applied together).

The changes in spectrochemical indices associated with the acyl chain of fatty acids (A_2922_/A_2955_), phosphorylation (A_1239_/A_2955_ and A_1080_/A_1545_), and carbonylation (A_1740_/A_1545_) of proteins^16^ are shown in Figure 5. Interestingly, IF and SCD Probiotics had the opposite effect on the acyl chain of fatty acids. There was no significant difference in the FPLI group where IF was combined with SCD Probiotics. While IF decreased the length of acyl chains, the treatment with SCD Probiotics increased their length (Figure 5A). Combining IF with SCD Probiotics significantly increased the spectrochemical markers of protein phosphorylation (Figure 5B,C). Furthermore, implementing IF is highly effective in substantially reducing protein carbonylation. Combining IF with SCD Probiotics might offer even more significant advantages in terms of this parameter (Figure 5D). The bandwidths of bands at 2922 cm^−1^ (CH_2_ antisymmetric stretching: lipids), 1653 cm^−1^ (Amide I: α‐helical structure of proteins), and spectrochemical index of membrane dynamics (Bw_2922_/Bw_2955_) also showed significant changes (Figure S4). The bandwidth value of the CH_2_ antisymmetric band located at 2922 cm^−1^ position decreased due to the combination of IF with SCD Probiotics (Figure S4a). The Amide I bandwidth at 1653 cm^−1^ position increased in the groups where the applications were administered individually (Figure S4b). Moreover, the index of membrane dynamics (Bw_2922_/Bw_2955_) improved separately in the IF and SCD Probiotics groups (Figure S4c).

### 3.3 Effects of IF, SCD probiotics and combination treatments on aged liver histology

**FIGURE 6:**
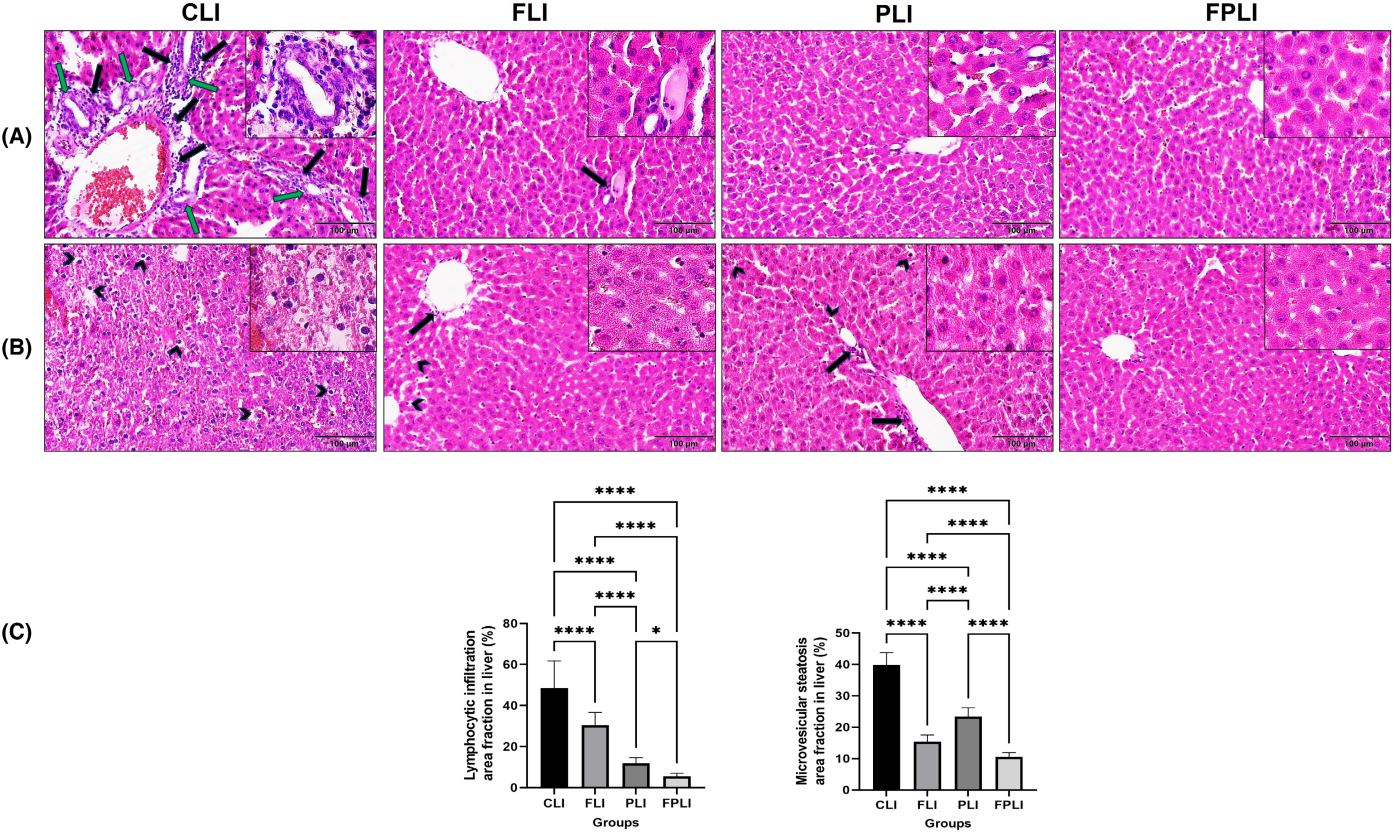
Aged rat livers show increased steatosis and lymphocytic infiltration, but IF, SCD probiotics, and combination treatment groups improved these histopathological alterations (A). Representative images of haematoxylin and eosin staining of lymphocytic infiltration and bile duct proliferation areas. Black arrows show lymphocytic infiltration and green arrows show bile ducts (B). Black arrow heads show hepatic microvesicular steatosis. Representative graphics of quantification of lymphocytic infiltration area fraction (%) and hepatic microvesicular steatosis area fraction (%) in all groups (C). The squared areas in the respective microphotographs of all groups are enlarged in the upper right corner. Values are expressed as mean ± SEM; *n* = 7 rats in each group. *p* < 0.05, and *p* ≤ 0.0001*** (nonparametric Mann–Whitney *U* test). Scale bar = 100 μm. CLI (control), FLI (Intermittent Fasting), PLI (SDC Probiotics), and the FPLI applications (in which the intermittent fasting and SCD Probiotics were applied together).

The study investigated the histopathological effects of IF SCD Probiotics and their combination on aged liver damage and fibrosis. As shown in Figure 6A–C, old control livers (CLI group) exhibited fibrosis, tissue damage, irregular hepatocyte arrangement, increased sinusoidal areas, increased Kupffer cells, and intense lymphocytic and neutrophil infiltration. In contrast, the FLI and PLI groups displayed normal and healthy liver architecture. IF and SCD Probiotics administration significantly improved hepatic fibrosis, cellular degeneration, and lymphocytic infiltration in all treated groups compared to the aged control group. A combination group of IF and SCD Probiotics appeared to reduce age‐related inflammation in the old liver. Additionally, bile duct proliferation decreased in the treated groups compared to the aged control group, indicating the potential of these treatments in alleviating age‐related liver damage and fibrosis.

### 3.4 Effects of IF, SCD probiotics and combination treatments on lipid droplet accumulation in aged rat hepatocytes

The study investigated the effects of IF, SCD probiotics and combination treatments on microvesicular steatosis, a form of hepatic fat deposition, in the aged‐related livers. Histological examination revealed hepatocyte nuclei condensation, nuclear membrane changes and fragmentation in the aged control group compared to all treated groups. However, IF, SCD Probiotics, and combination group exhibited a significant reduction in the density of fat droplets compared to the aged control group (Figure 6B). The administration of IF, SCD Probiotics and combination treatments to aged rats led to a substantial decrease in hepatic microvesicular steatosis, suggesting their potential in mitigating age‐related liver changes (Figure 6C).

### 3.5 IF, SCD probiotics and combination treatments decreases collagen accumulation of aged liver fibrosis

**FIGURE 7:**
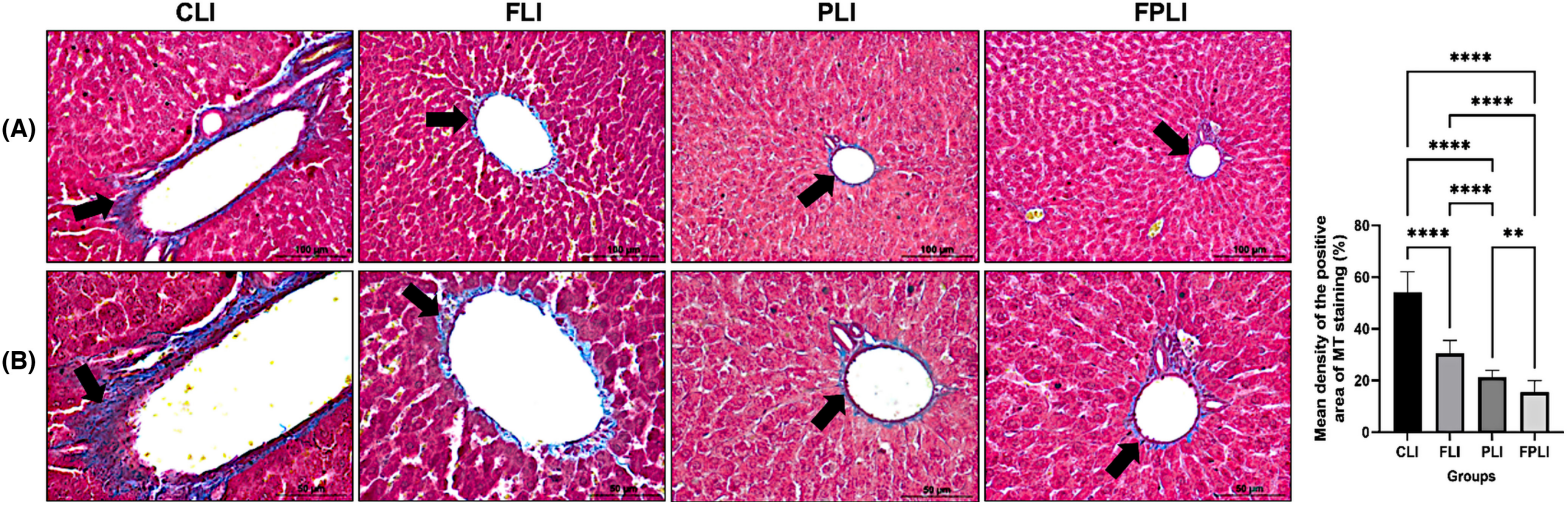
Representative images from MT staining depict collagen deposition in the liver tissues of aged control rats, as well as those treated with IF, SCD probiotics, and a combination of both treatments. The quantification of the collagen density area fraction (%) for all groups is also provided (A). Close‐up images of specific areas highlighted by black arrows in the microphotographs from the previous figure are displayed (B). Values are expressed as mean ± SEM; *n* = 7 rats in each group. *p* ≤ 0.01*, and *p* ≤ 0.0001*** (nonparametric Mann–Whitney *U* test) versus control. Scale bar = 50 and 100 μm. CLI (control), FLI (Intermittent Fasting), PLI (SDC Probiotics), and the FPLI applications (in which the intermittent fasting and SCD Probiotics were applied together).

To examine whether IF, SCD probiotics and combination treatments alleviate aging‐related liver fibrosis, collagen deposition was measured in the liver sections of all groups by using MT staining. The results showed that blue‐positive areas were significantly decreased in all treatment groups compared to the aged control group in Figure 7A,B shown with black arrows. However, compared to the aged control group, the levels of collagen deposition density were reduced considerably in the IF, SCD probiotics, and combination treatment groups, suggesting their potential to alleviate age‐related liver fibrosis (Figure 7).

### 3.6 Determination of biochemical liver enzymes in the serum

The results showed that serum ALP levels significantly increased in the SCD probiotics treatment group compared to the aged control group (Figure S5a). In contrast, ALT and AST levels showed no significant differences. There were no significant differences between the aged control and IF groups for ALP levels. ALP levels significantly increased in the SCD probiotics and combination treatment group compared to the IF group. However, ALP levels significantly decreased in the combination group compared to the SCD probiotics group.

Metabolic liver adaptations related to aging are poorly understood, but biochemical changes in LDH and albumin levels have been linked to liver diseases. LDH, an essential enzyme found primarily in muscle, liver, and kidney cells, showed increased serum levels in the IF, SCD probiotics, and combination treatment groups compared to the aged control group (Figure S5b). A decrease in serum albumin concentration with aging has been previously reported.^17^ Still, this study found that SCD probiotics and combination treatment with IF groups had significantly increased serum albumin levels compared to the aged control group (Figure S5c). In addition, serum albumin levels significantly increased in the SCD probiotics and combination treatment groups compared to the IF group.

## 4 DISCUSSION

During aging, the liver undergoes structural, functional and molecular changes that can impact its ability to carry out vital physiological processes, such as detoxification, metabolism and protein synthesis.^18^ Probiotics have been shown to modulate the gut‐liver axis, reduce oxidative stress and inflammation, and improve insulin sensitivity, which are essential factors in developing and progressing age‐related liver diseases.^19^ Recent evidence suggests that IF may also benefit liver health in aging individuals.^5^ Spectroscopy and machine learning analysis were utilized to analyse biomolecular profiles. In this study, machine learning and spectrochemical analyses evaluated the effects of co‐administration of IF and SCD Probiotics on the biomolecular structure of aged liver tissue. LDA analysis showed that IF and SCD Probiotics treatments had a specific impact on the lipid, protein, and nucleic biomolecule profiles but were more effective when applied together. Spectrochemical band analyses were performed to understand the observed differences better.

Aging can affect the metabolism and synthesis of cholesterol in the liver, which may further contribute to the accumulation of cholesterol. Spectral band analysis showed that both treatments significantly reduced the cholesterol ester value, formed when cholesterol, a type of lipid found in cell membranes and circulating in the bloodstream, is esterified with a fatty acid.^20^ Recent studies have suggested that probiotics supplementation may benefit cholesterol metabolism in aging individuals with liver dysfunction.^21^ Emerging evidence also suggests that IF may help cholesterol metabolism in aging individuals with liver dysfunction.^22^ However, they did not cause similar effects on the acyl chains of fatty acids. The acyl chain of fatty acids is a component of many lipids, including triglycerides and phospholipids, that play important roles in membrane structure and function, energy storage and cellular signalling.^23^ The most important effect of IF is the regeneration that it initiates at the cellular level. In this respect, we can say that more detailed research is needed to understand whether this value, which decreases after IF, or this value, which increases after SCD Probiotics application, is better because the application of both did not make a significant difference. However, it is seen that it may be more advantageous to do two applications at the same time to reduce the lipid ratio.

Combining both treatments enhanced protein phosphorylation, a process where a phosphate group is added to a protein molecule by a protein kinase enzyme, which may indicate cell regeneration. However, a complete understanding of the decrease or increase of this parameter also requires deeper analysis because this modification can regulate the activity of the protein, often by activating or deactivating it.^24^ The decrease in nucleic acid markers may also be considered an autophagy mechanism effect and may indicate cell regeneration. However, the fact that SCD Probiotics are more effective here also draws attention because it is reported that the activation of autophagy mechanisms requires a fasting state for a certain period.^25^ However, it is known that probiotic bacteria such as *Lactobacillus plantarum*, which is also included in SCD Probiotics, have the properties of activating the autophagy mechanisms.^26^ On the other hand, fully comprehending the differences in the Amid I and Amid II bands used to calculate protein content requires approaches such as proteomics.

The protein carbonylation rate is another critical spectrochemical band. It is one of the most harmful irreversible oxidative changes to proteins and is thought to be a significant sign of oxidative stress‐related disorders.^27^ Oxidative stress occurs when there is an imbalance between the production of reactive oxygen species and the body's ability to detoxify and repair the damage caused by these molecules. This process has been implicated in the development and progressing of various chronic diseases, including cardiovascular disease, cancer, and neurodegenerative disorders. Studies have shown that IF can induce the expression of antioxidant genes and increase the activity of antioxidant enzymes, which can help reduce oxidative stress and inflammation.^28^ This study observed that applying IF led to a notable reduction in protein carbonyl levels. Still, no significant alteration in this parameter was observed in the SCD Probiotics group. Nevertheless, the most substantial decrease in protein carbonylation was recorded when IF was combined with SCD Probiotics. IF application may affect the activation of the properties of probiotic bacteria in SCD Probiotics because it is known that *Bifidobacterium bifidum*, *Lactococcus lactis* and *Lactobacillus plantarum* species, which are also found in SCD Probiotics, play a role in reducing oxidative stress.^29^

Previous studies have shown that age‐related hyperinsulinemia can increase fat deposition, adipocyte size and lymphocytic infiltration, leading to hepatic fat deposition and systemic insulin resistance.^30^ In a study, researchers reported that the increase in microvesicular steatosis and lymphocytic infiltration in control‐aged mice might be associated with a decrease in age‐related hyperinsulinemia.^31^ Our study found that the IF, SCD probiotics and combination treatment groups significantly improved the increased microvesicular steatosis and lymphocytic infiltration compared to the aged control group. This curative effect may be related to the antioxidant and anti‐inflammatory activities of IF and probiotic administrations on liver functions.^32^ Combining IF with SCD Probiotics effectively reduced microvesicular steatosis and lymphocytic infiltration. Combined therapies in age‐related liver disorders can be an alternative medicine that protects and improves liver metabolism. Furthermore, reducing lymphocytic infiltration rates in treatment groups is assumed to improve hepatocyte inflammation due to aging. This is because of the decrease in fat deposits in the aged liver. Probiotics supplements and IF treatments have been previously found to effectively reduce body weight, fat content and cellular inflammation in old and diet‐induced obese mice.^33^, ^34^ Our results suggest that IF and SCD Probiotics may effectively prevent or reduce the severity of tissue damage in aging‐related metabolic diseases of the liver.

IF is considered one of the main physiological factors that can affect the aging liver.^35^ As liver function slightly declines in the elderly, various histological changes occur, such as collagen accumulation leading to liver fibrosis, typically associated with inflammation. Aging‐related liver fibrosis involves increased collagen accumulation, an extracellular matrix (ECM) component.^36^, ^37^ Our study found decreased collagen densities in IF, SCD Probiotics, and combined treatment groups compared to the aged control group, suggesting that IF and SCD Probiotics may help prevent age‐related liver fibrosis. This aligns with a study reporting IF's fibrosis reduction in the liver.^38^ Another study involving 15‐month‐old rats undergoing 3 months of IF revealed significant increases in liver damage, oxidative stress, inflammatory markers, hepatic vacuolations, cellular filtration, occlusion in central and portal veins, and collagen fibre surface area fraction in the elderly control group.^39^ These findings demonstrate that IF significantly reduces aging‐related histological changes in the liver. Our study, involving 24‐month‐old rats, suggests that IF may also reduce age‐related liver changes in later ages, differentiating it from the previous survey. IF's improving liver tissue inflammation by reducing lymphocytic infiltration and microvesicular steatosis supports its role in modulating autophagy in aged liver tissue. Combining IF and SCD Probiotics supplementation may offer a new therapeutic approach for geriatric liver intervention. Moreover, IF and natural probiotic applications could be a simple, reliable, healthy and cost‐effective treatment strategy for preventing changes in the aged liver.

Low ALP, LDH and albumin levels are associated with decreased liver metabolic function in aging and may correlate with age‐related chronic diseases.^40^ A previous study showed that aging‐related decreases in the aspartate‐alanine aminotransferase ratio could predict non‐alcoholic fatty liver disease and advanced fibrosis.^41^ Our recent publication demonstrated that young plasma administration increased ALP, LDH and albumin levels in aged rats, indicating improved liver function.^42^ This study found significant increases in LDH and albumin levels in IF, SCD Probiotic and combined supplementation groups compared to the control group. Age‐related muscle and bone mass decreases might contribute to lower LDH levels.^43^, ^44^ Additionally, changes in ALP levels, an enzyme associated with liver, kidney, and bone tissue, might affect aging. We found increased ALP activity in the IF, SCD Probiotics and combined supplementation groups compared to the control group. Age‐related changes in intestinal functions and microflora could cause a decrease in ALP levels. In aged rats, IF, SCD Probiotics and combined applications may have demonstrated anti‐inflammatory effects on both intestinal and liver functions, resulting in anti‐aging benefits. Increased metabolic activity due to IF and SCD Probiotics supplementation might have contributed to higher liver enzyme levels in elderly individuals. Additionally, lower LDH activity in older adults has been reported to be associated with lower total protein content related to aging.^43^, ^44^ Our findings are consistent with previously reported research data.

In conclusion, this study demonstrates the potential benefits of SCD Probiotics supplementation in combination with a 30‐day IF program on liver biomolecule content and histological changes in aged male Sprague–Dawley rats. The findings reveal significant alterations in lipid, protein, and nucleic acid profiles with individual or combined treatments. LDA and SVM machine learning techniques accurately differentiated the control and treated groups. The combined application of IF and SCD Probiotics resulted in the most significant improvements, including reduced cholesterol ester levels and enhanced protein phosphorylation and carbonylation. However, individual treatments of IF and SCD Probiotics were more effective in improving membrane dynamics. Histological evaluations showed decreased hepatocyte degeneration, lymphocytic infiltration, steatosis and fibrosis in the treated groups. Moreover, serum ALP, LDH and albumin levels increased significantly in the SCD Probiotics and combined treatment groups. These results provide valuable insights into the underlying mechanisms behind the beneficial effects of IF and SCD Probiotics supplementation on biomolecule content in liver tissue. The findings may contribute to developing personalized nutrition and health strategies for aging individuals. Further research is needed to explore these treatments' long‐term effects and investigate their potential applications in humans.

## AUTHOR CONTRIBUTIONS

**Hikmet Taner Teker:** Conceptualization (equal); data curation (equal); formal analysis (equal); funding acquisition (equal); investigation (equal); methodology (equal); resources (equal); software (equal); supervision (equal); validation (equal); visualization (equal); writing – original draft (equal); writing – review and editing (equal). **Taha Ceylani:** Conceptualization (equal); data curation (equal); formal analysis (equal); funding acquisition (equal); investigation (equal); methodology (equal); resources (equal); software (equal); supervision (equal); validation (equal); visualization (equal); writing – original draft (equal); writing – review and editing (equal). **Seda Keskin:** Methodology (equal); resources (equal); software (equal); supervision (equal); validation (equal); visualization (equal); writing – original draft (equal); writing – review and editing (equal). **Gizem Samgane:** Methodology (equal); resources (equal); software (equal); supervision (equal); validation (equal); visualization (equal). **Burcu Baba:** Investigation (equal); methodology (equal); resources (equal); software (equal); supervision (equal); validation (equal); visualization (equal). **Eda Acıkgoz:** Methodology (equal); resources (equal); software (equal); supervision (equal); validation (equal); visualization (equal); writing – original draft (equal); writing – review and editing (equal). **Rafig Gurbanov:** Investigation (equal); methodology (equal); project administration (equal); resources (equal); software (equal); supervision (equal); validation (equal); visualization (equal); writing – original draft (equal); writing – review and editing (equal).

## FUNDING INFORMATION

This research received no specific grant from any funding agency in the public, commercial, or not‐for‐profit sectors.

## CONFLICT OF INTEREST STATEMENT

The authors have declared that no competing interests exist.

## ETHICS STATEMENT

The animals were kept according to standard animal care procedures, and the study was approved by the Ethics Committee (permission number: 2021/05) of the Saki Yenilli Experimental Animal Production and Practice Laboratory.

## CONSENT FOR PUBLICATION

Not applicable.

## Supporting information


Figure S1.

Table S1.

Data S1.

## Data Availability

The datasets generated during and/or analysed during the current study are available from the corresponding author upon reasonable request.
