# Supplementary material for: Reduced liver damage and fibrosis with combined SCD Probiotics and intermittent fasting in aged rat
Source: J Cell Mol Med. 2023 Oct 28;28(1):e18014. doi: 10.1111/jcmm.18014 (PMC10805504; doi:10.1111/jcmm.18014)
Supplement: Supplementary file 1 — Figure S1. [file JCMM-28-e18014-s002.docx]

**SUPPLEMENTARY FIGURES**

**Reduced liver damage and fibrosis with combined SCD Probiotics and intermittent fasting in aged rat**

Hikmet Taner Teker^1^, Taha Ceylani^2,3*^, Seda Keskin^4^, Gizem Samgane^5^, Burcu Baba^6^, Eda Acıkgoz^4^, and Rafig Gurbanov^5,7*^

^1^ Department of Medical Biology and Genetics, Ankara Medipol University Ankara, Turkey ^2^ Department of Molecular Biology and Genetics, Muş Alparslan University Muş, Turkey

^3^ Department of Food Quality Control and Analysis, Muş Alparslan University Muş, Turkey

^4^ Department of Histology and Embryology, Van Yuzuncu Yil University, Van, Turkey

^5^ Department of Bioengineering, Bilecik Şeyh Edebali University Bilecik, Turkey

^6^ Department of Medical Biochemistry, Yüksek İhtisas University, Ankara, Turkey

^7^ Central Research Laboratory (BARUM), Bilecik Şeyh Edebali University Bilecik, Turkey

* Correspondence: [rafig.gurbanov@bilecik.edu.tr](mailto:rafig.gurbanov@bilecik.edu.tr), t.ceylani@alparslan.edu.tr

ORCID ID: 0000-0002-5293-6447 (R. Gurbanov)

ORCID ID: 0000-0002-3041-6010 (T. Ceylani)

**
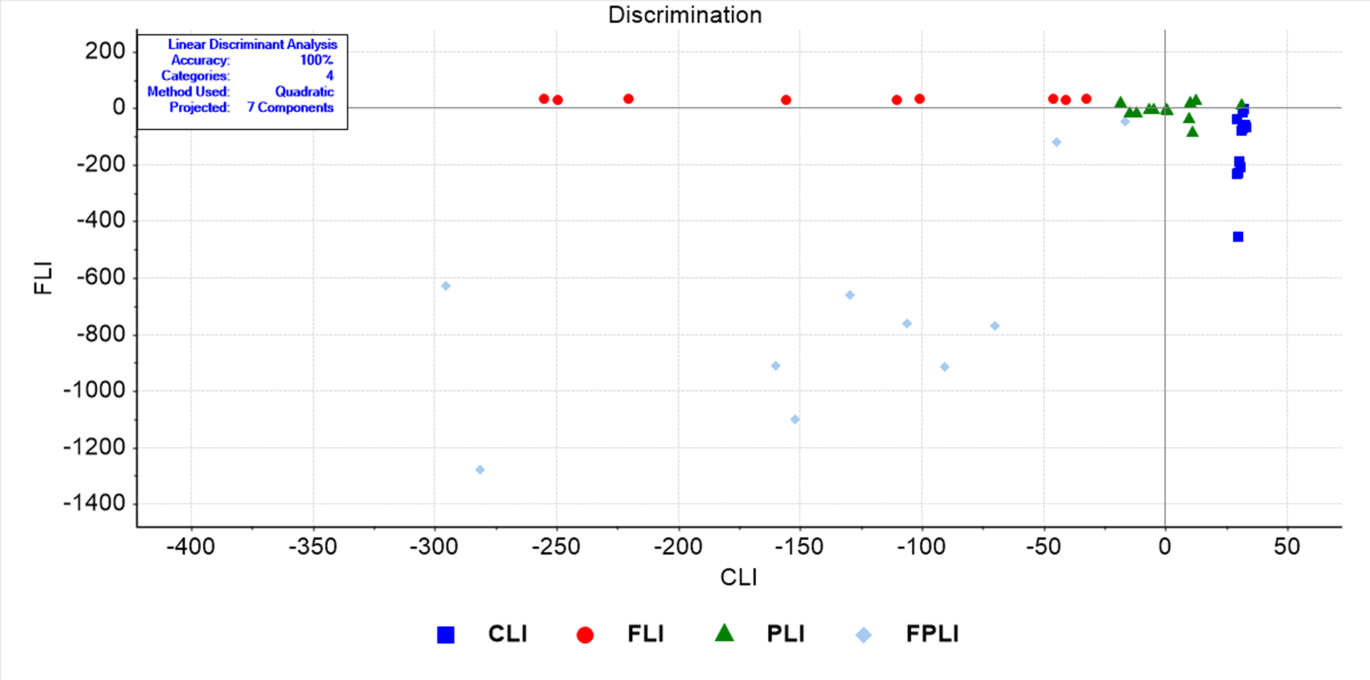
**

**Fig. S1** LDA discrimination plot for liver samples in protein (1700-1500 cm^-1^) spectral region. CLI (control), FLI (Intermittent Fasting), PLI (SDC Probiotics), and the FPLI applications (in which the intermittent fasting and SCD Probiotics were applied together).

**
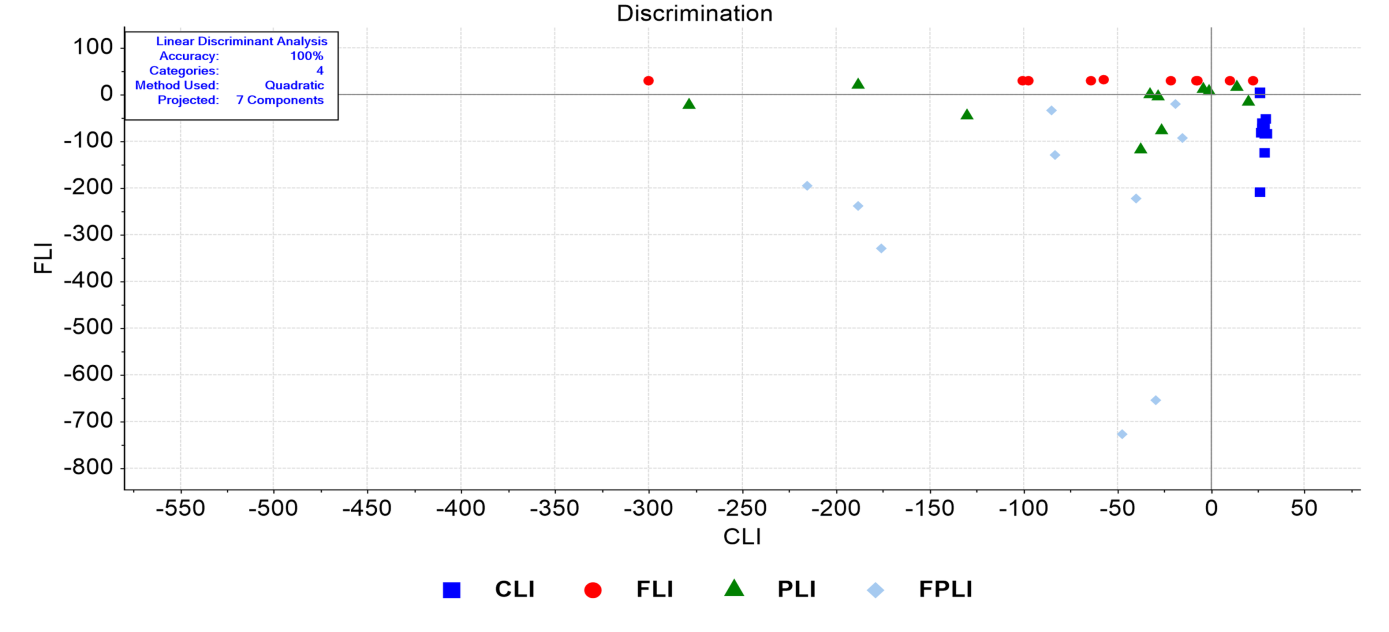
**

**Fig. S2** LDA discrimination plot for liver samples in nucleic acids and polysaccharides (1200-650 cm^-1^) spectral region. CLI (control), FLI (Intermittent Fasting), PLI (SDC Probiotics), and the FPLI applications (in which the intermittent fasting and SCD Probiotics were applied together).


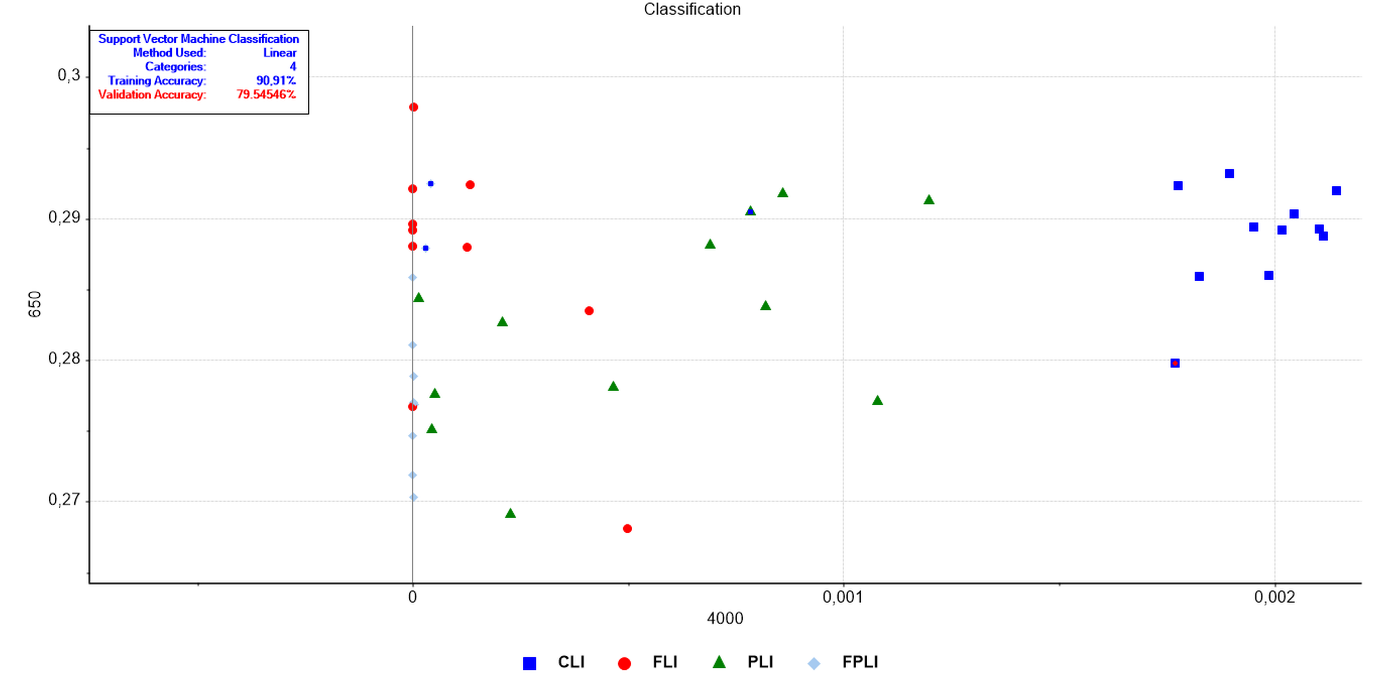


**Fig. S3.** SVM classification plot for liver samples in the full (4000-650 cm^-1^) spectral region. CLI (control), FLI (Intermittent Fasting), PLI (SDC Probiotics), and the FPLI applications (in which the intermittent fasting and SCD Probiotics were applied together).


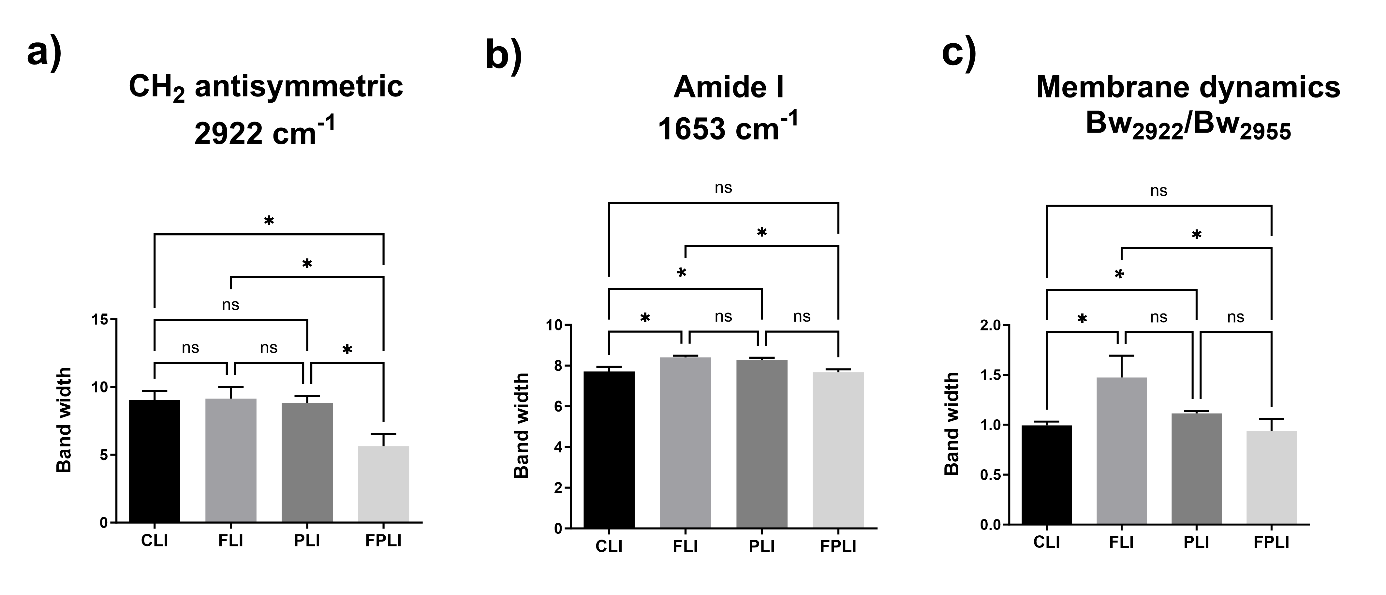


**Fig. S4** The quantitative changes in bandwidth parameters. The indices for **a)** 2922 cm^-1^ (CH_2_ antisymmetric stretching: lipids and proteins), **b)** 1653 cm^-1^ (Amide I: *α-*helical structure of proteins), and **c)** Membrane dynamics (Bw_2922_/Bw_2955_), CLI (control), FLI (Intermittent Fasting), PLI (SDC Probiotics), and the FPLI applications (in which the intermittent fasting and SCD Probiotics were applied together).


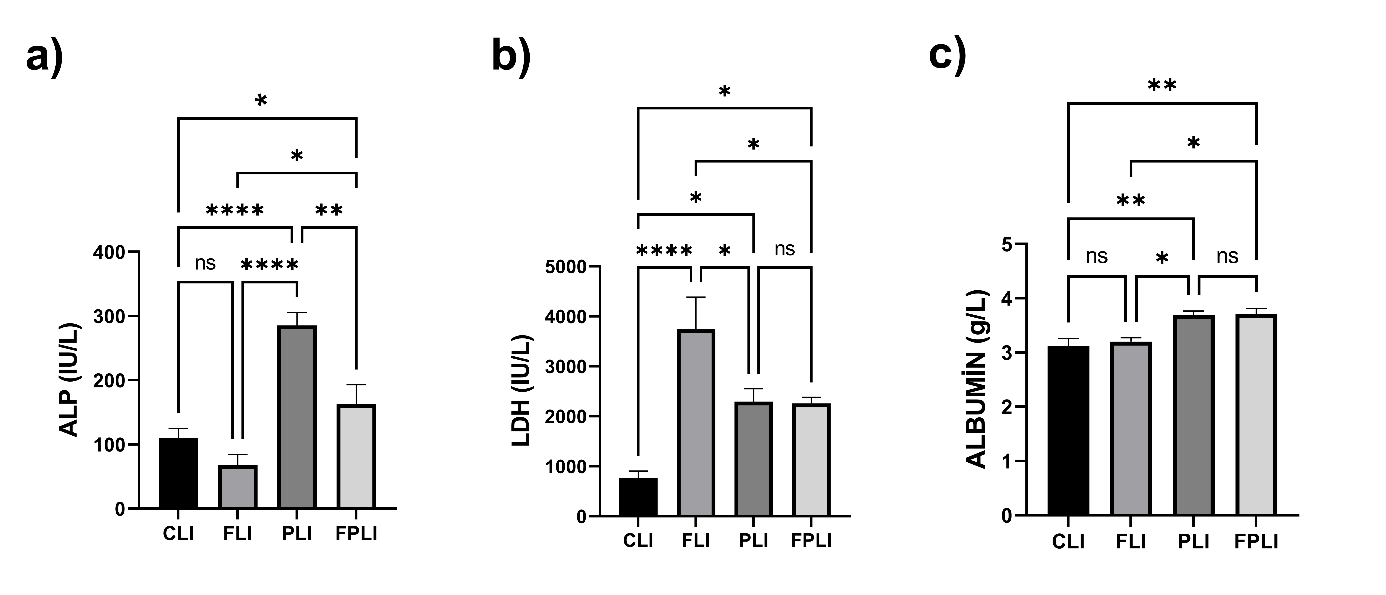
**Fig S5** Effects of all treatment groups (FLI, PLI, and FPLI groups) on the serum levels of **a)** ALP, **b)** LDH, and **c)** Albumin. Values were expressed as mean ± SEM, n = 7 group. *P < 0.05 and P ≤ 0.01 ** vs. control. CLI (control), FLI (Intermittent Fasting), PLI (SDC Probiotics), and the FPLI applications (in which the intermittent fasting and SCD Probiotics were applied together).
