# Supplementary material for: Reduced liver damage and fibrosis with combined SCD Probiotics and intermittent fasting in aged rat
Source: J Cell Mol Med. 2023 Oct 28;28(1):e18014. doi: 10.1111/jcmm.18014 (PMC10805504; doi:10.1111/jcmm.18014)
Supplement: Supplementary file 2 — Table S1. [file JCMM-28-e18014-s003.docx]

**SUPLEMENTARY TABLES**

**Reduced liver damage and fibrosis with combined SCD Probiotics and intermittent fasting in aged rat**

Hikmet Taner Teker^1^, Taha Ceylani^2-3*^, Seda Keskin^4^, Gizem Samgane^5^, Burcu Baba^6^, Eda Acıkgoz^4^, and Rafig Gurbanov^5,7*^

^1^ Department of Medical Biology and Genetics, Ankara Medipol University Ankara, Turkey ^2^ Department of Molecular Biology and Genetics, Muş Alparslan University Muş, Turkey

^3^ Department of Food Quality Control and Analysis, Muş Alparslan University Muş, Turkey

^4^ Department of Histology and Embryology, Van Yuzuncu Yil University, Van, Turkey

^5^ Department of Bioengineering, Bilecik Şeyh Edebali University Bilecik, Turkey

^6^ Department of Medical Biochemistry, Yüksek İhtisas University, Ankara, Turkey

^7^ Central Research Laboratory, Bilecik Şeyh Edebali University Bilecik, Turkey

* Correspondence: rafig.gurbanov@bilecik.edu.tr, t.ceylani@alparslan.edu.tr

ORCID ID: 0000-0002-5293-6447 (R. Gurbanov)

ORCID ID: 0000-0002-3041-6010 (T. Ceylani)

**Supplementary Tables**

**Table S1** LDA confusion matrix for liver samples in the full (4000-650 cm^-1^) spectral region. CLI (control) and FLI (intermittent fasting), PLI (SCD Probiotics) and FPLI (in which the IF and SCD Probiotics).

| **Confusion matrix** | **Actual** | **CLI** | **FLI** | **PLI** | **FPLI** |
| --- | --- | --- | --- | --- | --- |
| Predicted |  | 1 | 2 | 3 | 4 |
| **CLI** | 1 | **12** | 0 | 0 | 0 |
| **FLI** | 2 | 0 | **10** | 0 | 0 |
| **PLI** | 3 | 0 | 0 | **12** | 0 |
| **FPLI** | 4 | 0 | 0 | 0 | **10** |

**Table S2** LDA confusion matrix for liver samples in the full (4000-650 cm^-1^) spectral region. CLI (control) and FLI (intermittent fasting), PLI (SCD Probiotics) and FPLI (in which the IF and SCD Probiotics).

|  | **CIL** | **FIL** | **PIL** | **FPIL** | **Predicted** |
| --- | --- | --- | --- | --- | --- |
|  | 1 | 2 | 3 | 4 | 5 |
| 1 | 19,31 | 6,57 | -2,91 | -416,36 | CLI |
| 2 | 21,11 | -47,11 | 10,73 | -279,33 | CLI |
| 3 | 21,53 | -63,54 | -1,41 | -373,24 | CLI |
| 4 | 22,15 | -58,76 | 2,69 | -408,55 | CLI |
| 5 | 20,86 | -27,04 | -11,45 | -346,30 | CLI |
| 6 | 20,82 | -51,57 | -101,68 | -528,89 | CLI |
| 7 | 23,06 | -29,16 | -1,02 | -228,84 | CLI |
| 8 | 20,87 | -57,27 | -4,97 | -237,61 | CLI |
| 9 | 20,65 | -0,96 | -3,81 | -247,77 | CLI |
| 10 | 22,03 | -24,51 | 1,73 | -303,45 | CLI |
| 11 | 19,42 | -83,62 | -356,98 | -619,19 | CLI |
| 12 | 21,01 | -14,29 | -30,73 | -297,09 | CLI |
| 13 | -95,32 | 20,42 | -2,76 | -238,61 | FLI |
| 14 | 10,35 | 20,86 | 3,22 | -398,76 | FLI |
| 15 | -19,70 | 20,63 | -13,38 | -623,69 | FLI |
| 16 | -126,98 | 21,14 | -5,94 | -918,81 | FLI |
| 17 | -74,36 | 21,05 | 6,06 | -209,65 | FLI |
| 18 | -68,95 | 23,78 | 4,86 | -318,36 | FLI |
| 19 | -103,97 | 20,95 | 2,11 | -229,96 | FLI |
| 20 | -309,29 | 20,38 | -56,61 | -300,86 | FLI |
| 21 | -31,58 | 22,64 | -3,81 | -484,43 | FLI |
| 22 | -50,76 | 20,59 | 9,85 | -286,49 | FLI |
| 23 | -46,94 | -18,56 | 18,60 | -383,42 | PLI |
| 24 | 9,07 | -22,27 | 19,21 | -415,78 | PLI |
| 25 | 3,95 | 12,95 | 18,84 | -422,18 | PLI |
| 26 | 8,58 | 2,63 | 22,60 | -425,25 | PLI |
| 27 | -52,17 | -10,87 | 19,71 | -227,91 | PLI |
| 28 | -12,75 | -3,77 | 20,68 | -329,28 | PLI |
| 29 | -35,26 | -4,20 | 22,08 | -456,53 | PLI |
| 30 | -56,19 | -27,87 | 19,12 | -514,05 | PLI |
| 31 | -153,32 | -130,25 | 18,35 | -563,81 | PLI |
| 32 | -69,61 | -7,28 | 21,80 | -694,67 | PLI |
| 33 | -195,16 | -59,81 | 18,64 | -1243,25 | PLI |
| 34 | -147,18 | -22,32 | 19,13 | -1004,93 | PLI |
| 35 | -614,76 | -68,43 | -42,90 | 19,99 | FPLI |
| 36 | -959,88 | -189,41 | -57,72 | 19,97 | FPLI |
| 37 | -295,76 | -215,89 | -34,01 | 20,78 | FPLI |
| 38 | -399,14 | -391,19 | -56,06 | 20,16 | FPLI |
| 39 | -307,53 | -319,06 | -55,81 | 20,56 | FPLI |
| 40 | -366,63 | -403,96 | -76,14 | 22,64 | FPLI |
| 41 | -306,91 | -272,73 | -92,61 | 19,72 | FPLI |
| 42 | -552,46 | -764,21 | -152,57 | 19,70 | FPLI |
| 43 | -285,33 | -362,49 | -108,88 | 21,00 | FPLI |
| 44 | -349,39 | -318,37 | -65,58 | 21,23 | FPLI |

**Table S3** LDA confusion matrix for liver samples in lipid (3000-2700 cm^-1^) spectral region. CLI (control) and FLI (intermittent fasting), PLI (SCD Probiotics) and FPLI (in which the IF and SCD Probiotics).

| **Confusion matrix** | **Actual** | **CLI** | **FLI** | **PLI** | **FPLI** |
| --- | --- | --- | --- | --- | --- |
| Predicted |  | 1 | 2 | 3 | 4 |
| **CLI** | 1 | **12** | 0 | 0 | 0 |
| **FLI** | 2 | 0 | **9** | 0 | 0 |
| **PLI** | 3 | 0 | 1 | **12** | 0 |
| **FPLI** | 4 | 0 | 0 | 0 | **10** |

**Table S4** LDA confusion matrix for liver samples in lipid (3000-2700 cm^-1^) spectral region. CLI (control) and FLI (intermittent fasting), PLI (SCD Probiotics) and FPLI (in which the IF and SCD Probiotics).

|  | **CLI** | **FLI** | **PLI** | **FPLI** | **Predicted** |
| --- | --- | --- | --- | --- | --- |
|  | 1 | 2 | 3 | 4 | 5 |
| 1 | 39,59 | 36,43 | 11,68 | -170,13 | CLI |
| 2 | 40,64 | -43,03 | 15,99 | 38,16 | CLI |
| 3 | 42,11 | -27,81 | -8,62 | 7,48 | CLI |
| 4 | 42,41 | -26,37 | 5,91 | 18,57 | CLI |
| 5 | 40,05 | 2,46 | -23,65 | -32,56 | CLI |
| 6 | 40,63 | -4,21 | -30,20 | -13,34 | CLI |
| 7 | 42,21 | -40,59 | -4,28 | 18,83 | CLI |
| 8 | 43,33 | -59,87 | -12,72 | 27,14 | CLI |
| 9 | 41,97 | 9,60 | 4,42 | -10,67 | CLI |
| 10 | 42,94 | -31,59 | -9,12 | 15,56 | CLI |
| 11 | 39,61 | -79,93 | -247,06 | -378,84 | CLI |
| 12 | 41,25 | -15,08 | -5,47 | -18,81 | CLI |
| 13 | -14,89 | 40,08 | 14,10 | -145,85 | FLI |
| 14 | 28,43 | 39,86 | 41,06 | 32,49 | PLI |
| 15 | 29,96 | 40,67 | 20,11 | -51,26 | FLI |
| 16 | -75,11 | 39,83 | 32,43 | -19,94 | FLI |
| 17 | 12,10 | 40,50 | 12,35 | -77,28 | FLI |
| 18 | 2,78 | 42,89 | 25,21 | -51,37 | FLI |
| 19 | 8,77 | 41,88 | 24,55 | -87,94 | FLI |
| 20 | -67,73 | 39,99 | -24,12 | -319,65 | FLI |
| 21 | -122,84 | 40,12 | -30,78 | -116,95 | FLI |
| 22 | -104,55 | 40,92 | 24,27 | -100,14 | FLI |
| 23 | -21,01 | 18,22 | 39,96 | 21,83 | PLI |
| 24 | -1,03 | 8,21 | 40,13 | 38,73 | PLI |
| 25 | 20,31 | 36,86 | 40,37 | 29,73 | PLI |
| 26 | 3,72 | 25,27 | 40,54 | 5,49 | PLI |
| 27 | 8,66 | 27,81 | 39,57 | 4,75 | PLI |
| 28 | 24,07 | 27,29 | 43,76 | 15,99 | PLI |
| 29 | -1,48 | 19,99 | 42,90 | -43,09 | PLI |
| 30 | 6,26 | -2,60 | 42,46 | -60,88 | PLI |
| 31 | -28,29 | -33,99 | 40,65 | -183,33 | PLI |
| 32 | 2,25 | 14,80 | 42,78 | -97,60 | PLI |
| 33 | -71,86 | -38,86 | 40,99 | -187,54 | PLI |
| 34 | -53,68 | -13,22 | 41,69 | -133,05 | PLI |
| 35 | 37,00 | -4,31 | 17,74 | 42,39 | FPLI |
| 36 | 40,68 | -42,73 | 21,74 | 42,33 | FPLI |
| 37 | -201,11 | -13,43 | 11,24 | 41,06 | FPLI |
| 38 | -204,04 | -115,91 | 10,11 | 42,70 | FPLI |
| 39 | -197,12 | -107,27 | 4,85 | 40,71 | FPLI |
| 40 | -199,44 | -189,47 | -1,41 | 42,96 | FPLI |
| 41 | -160,03 | -19,77 | 28,02 | 41,01 | FPLI |
| 42 | -248,66 | -27,44 | -82,18 | 40,73 | FPLI |
| 43 | -232,49 | -167,19 | 1,08 | 40,85 | FPLI |
| 44 | -273,24 | -113,81 | -13,31 | 41,22 | FPLI |

**Table S5** LDA confusion matrix for liver samples in protein (1700-1500 cm^-1^) spectral region. CLI (control) and FLI (intermittent fasting), PLI (SCD Probiotics) and FPLI (in which the IF and SCD Probiotics).

| **Confusion matrix** | **Actual** | **CLI** | **FLI** | **PLI** | **FPLI** |
| --- | --- | --- | --- | --- | --- |
| Predicted |  | 1 | 2 | 3 | 4 |
| **CLI** | 1 | **12** | 0 | 0 | 0 |
| **FLI** | 2 | 0 | **10** | 0 | 0 |
| **PLI** | 3 | 0 | 0 | **12** | 0 |
| **FPLI** | 4 | 0 | 0 | 0 | **10** |

**Table S6** LDA confusion matrix for liver samples in protein (1700-1500 cm^-1^) spectral region. CLI (control) and FLI (intermittent fasting), PLI (SCD Probiotics) and FPLI (in which the IF and SCD Probiotics).

|  | CLI | FLI | PLI | FPLI | Predicted |
| --- | --- | --- | --- | --- | --- |
|  | 1 | 2 | 3 | 4 | 5 |
| 1 | 29,17 | -39,69 | 18,36 | -913,70 | CLI |
| 2 | 31,69 | -69,22 | 27,22 | -51,36 | CLI |
| 3 | 31,75 | -12,94 | 20,81 | -305,41 | CLI |
| 4 | 32,00 | -3,24 | 23,64 | -207,20 | CLI |
| 5 | 30,61 | -206,43 | -13,45 | -705,41 | CLI |
| 6 | 29,71 | -454,95 | -48,57 | -759,45 | CLI |
| 7 | 31,38 | -77,92 | 24,40 | -103,29 | CLI |
| 8 | 32,72 | -60,47 | 28,68 | -65,70 | CLI |
| 9 | 30,37 | -186,85 | 9,02 | -344,19 | CLI |
| 10 | 33,10 | -66,80 | 28,91 | -122,48 | CLI |
| 11 | 29,20 | -232,15 | -281,22 | -1406,95 | CLI |
| 12 | 29,83 | -228,69 | -1,77 | -305,17 | CLI |
| 13 | -46,16 | 31,90 | 10,67 | -501,20 | FLI |
| 14 | -32,61 | 33,29 | 29,74 | -32,87 | FLI |
| 15 | -40,94 | 31,49 | 16,53 | -425,44 | FLI |
| 16 | -249,40 | 31,28 | 21,09 | -969,36 | FLI |
| 17 | -255,05 | 33,40 | 17,65 | -59,61 | FLI |
| 18 | -220,55 | 31,96 | 20,26 | -102,38 | FLI |
| 19 | -110,32 | 31,60 | 11,21 | -36,18 | FLI |
| 20 | -383,00 | 31,42 | -62,44 | -352,48 | FLI |
| 21 | -101,16 | 31,86 | 26,71 | -103,34 | FLI |
| 22 | -155,76 | 31,40 | 23,91 | -111,68 | FLI |
| 23 | -14,72 | -19,33 | 29,27 | -152,19 | PLI |
| 24 | -11,98 | -19,03 | 30,30 | -242,14 | PLI |
| 25 | -18,53 | 18,86 | 29,39 | -29,44 | PLI |
| 26 | 12,57 | 23,84 | 32,24 | -74,37 | PLI |
| 27 | -4,93 | -4,87 | 30,39 | -330,34 | PLI |
| 28 | 31,37 | 8,39 | 31,53 | -158,50 | PLI |
| 29 | 10,13 | 17,73 | 31,46 | -156,86 | PLI |
| 30 | 0,22 | -5,28 | 29,81 | -206,27 | PLI |
| 31 | 9,56 | -38,98 | 29,09 | -1253,21 | PLI |
| 32 | -6,63 | -5,83 | 29,69 | -1188,20 | PLI |
| 33 | 11,02 | -86,95 | 28,83 | -1510,16 | PLI |
| 34 | 0,59 | -10,18 | 28,76 | -1394,94 | PLI |
| 35 | -44,76 | -119,17 | 22,12 | 31,64 | FPLI |
| 36 | -16,73 | -48,06 | 18,71 | 31,95 | FPLI |
| 37 | -281,52 | -1278,15 | -94,78 | 31,07 | FPLI |
| 38 | -152,14 | -1101,62 | -29,46 | 31,48 | FPLI |
| 39 | -129,65 | -662,54 | -16,58 | 31,48 | FPLI |
| 40 | -159,80 | -911,02 | -26,79 | 32,41 | FPLI |
| 41 | -106,03 | -759,56 | -31,51 | 32,11 | FPLI |
| 42 | -295,32 | -629,11 | -62,06 | 30,93 | FPLI |
| 43 | -90,64 | -915,11 | -24,41 | 31,02 | FPLI |
| 44 | -70,07 | -770,24 | -15,26 | 34,02 | FPLI |

**Table S7** LDA confusion matrix for liver samples in spectral region in nucleic acids and polysaccharides (1200-650 cm^-1^). CLI (control) and FLI (intermittent fasting), PLI (SCD Probiotics) and FPLI (in which the IF and SCD Probiotics).

| **Confusion matrix** | **Actual** | **CLI** | **FLI** | **PLI** | **FPLI** |
| --- | --- | --- | --- | --- | --- |
| Predicted |  | 1 | 2 | 3 | 4 |
| **CLI** | 1 | **12** | 0 | 0 | 0 |
| **FLI** | 2 | 0 | **10** | 0 | 0 |
| **PLI** | 3 | 0 | 0 | **12** | 0 |
| **FPLI** | 4 | 0 | 0 | 0 | **10** |

**Table S8** LDA confusion matrix for liver samples in spectral region in nucleic acids and polysaccharides (1200-650 cm^-1^). CLI (control) and FLI (intermittent fasting), PLI (SCD Probiotics) and FPLI (in which the IF and SCD Probiotics).

|  | **CLI** | **FLI** | **PLI** | **FPLI** | **Predicted** |
| --- | --- | --- | --- | --- | --- |
|  | 1 | 2 | 3 | 4 | 5 |
| 1 | 26,21 | 1,76 | 20,71 | -92,24 | CLI |
| 2 | 26,16 | 4,52 | 23,96 | 18,85 | CLI |
| 3 | 27,73 | -62,75 | -92,07 | -60,74 | CLI |
| 4 | 28,90 | -70,24 | -92,52 | -25,89 | CLI |
| 5 | 28,11 | -68,92 | -143,05 | 14,90 | CLI |
| 6 | 28,84 | -125,27 | -176,29 | 20,97 | CLI |
| 7 | 29,96 | -84,37 | -51,19 | 23,08 | CLI |
| 8 | 26,96 | -82,02 | -33,71 | 18,79 | CLI |
| 9 | 29,50 | -52,56 | -71,81 | 15,27 | CLI |
| 10 | 27,77 | -73,16 | -63,56 | 23,02 | CLI |
| 11 | 26,35 | -208,15 | -276,00 | -45,27 | CLI |
| 12 | 28,58 | -84,91 | -106,60 | 7,54 | CLI |
| 13 | -97,31 | 28,76 | -5,14 | -22,46 | FLI |
| 14 | 22,75 | 29,57 | 21,66 | 9,57 | FLI |
| 15 | -21,27 | 28,36 | 15,02 | -33,01 | FLI |
| 16 | -300,06 | 28,37 | 20,38 | -44,21 | FLI |
| 17 | -7,19 | 29,29 | 18,81 | -16,17 | FLI |
| 18 | -57,34 | 31,66 | 18,55 | -18,67 | FLI |
| 19 | -7,88 | 29,17 | 10,71 | -11,78 | FLI |
| 20 | 10,08 | 28,73 | -30,63 | -73,81 | FLI |
| 21 | -100,41 | 29,56 | 18,14 | -22,36 | FLI |
| 22 | -63,77 | 28,29 | 17,85 | -76,39 | FLI |
| 23 | -129,87 | -44,61 | 26,53 | -32,29 | PLI |
| 24 | 19,92 | -15,37 | 26,60 | 12,72 | PLI |
| 25 | -28,35 | -4,78 | 27,54 | -4,80 | PLI |
| 26 | -4,29 | 12,01 | 28,49 | -20,60 | PLI |
| 27 | -0,72 | 6,96 | 29,40 | -17,67 | PLI |
| 28 | 13,71 | 14,60 | 27,28 | 6,14 | PLI |
| 29 | -32,42 | -0,59 | 29,19 | -40,89 | PLI |
| 30 | -26,34 | -78,34 | 28,30 | -38,01 | PLI |
| 31 | -37,31 | -117,71 | 26,68 | -143,75 | PLI |
| 32 | -188,35 | 20,46 | 29,65 | -129,18 | PLI |
| 33 | -540,34 | -13,20 | 26,96 | -216,45 | PLI |
| 34 | -278,33 | -22,46 | 26,59 | -104,41 | PLI |
| 35 | -29,62 | -653,24 | -780,91 | 27,27 | FPLI |
| 36 | -47,03 | -727,48 | -597,69 | 27,64 | FPLI |
| 37 | -84,72 | -34,17 | -24,92 | 26,73 | FPLI |
| 38 | -188,37 | -238,63 | -29,26 | 27,07 | FPLI |
| 39 | -215,41 | -196,57 | -28,29 | 26,44 | FPLI |
| 40 | -176,00 | -329,67 | -25,59 | 28,06 | FPLI |
| 41 | -19,06 | -20,17 | -1,32 | 28,08 | FPLI |
| 42 | -15,21 | -94,26 | -3,18 | 26,46 | FPLI |
| 43 | -39,88 | -221,95 | -65,79 | 27,07 | FPLI |
| 44 | -83,04 | -129,62 | -96,69 | 28,39 | FPLI |
