# Supplementary material for: Reduced liver damage and fibrosis with combined SCD Probiotics and intermittent fasting in aged rat
Source: J Cell Mol Med. 2023 Oct 28;28(1):e18014. doi: 10.1111/jcmm.18014 (PMC10805504; doi:10.1111/jcmm.18014)
Supplement: Supplementary file 3 — Data S1. [file JCMM-28-e18014-s001.docx]

**MATERIALS AND METHODS**

**Reduced liver damage and fibrosis with combined SCD Probiotics and intermittent fasting in aged rat**

Hikmet Taner Teker^1^, Taha Ceylani^2,3*^, Seda Keskin^4^, Gizem Samgane^5^, Burcu Baba^6^, Eda Acıkgoz^4^, and Rafig Gurbanov^5,7*^

^1^ Department of Medical Biology and Genetics, Ankara Medipol University Ankara, Turkey ^2^ Department of Molecular Biology and Genetics, Muş Alparslan University Muş, Turkey

^3^ Department of Food Quality Control and Analysis, Muş Alparslan University Muş, Turkey

^4^ Department of Histology and Embryology, Van Yuzuncu Yil University, Van, Turkey

^5^ Department of Bioengineering, Bilecik Şeyh Edebali University Bilecik, Turkey

^6^ Department of Medical Biochemistry, Yüksek İhtisas University, Ankara, Turkey

^7^ Central Research Laboratory (BARUM), Bilecik Şeyh Edebali University Bilecik, Turkey

* Correspondence: rafig.gurbanov@bilecik.edu.tr, t.ceylani@alparslan.edu.tr

ORCID ID: 0000-0002-5293-6447 (R. Gurbanov)

ORCID ID: 0000-0002-3041-6010 (T. Ceylani)

**2. MATERIALS AND METHODS**

**2.1 Animal studies**

This study used male Sprague-Dawley rats (24 months old) as the model organism. The animals were divided into four groups: the control group (n=7), the intermittent fasting group (n=7), the SCD Probiotics group (n=7), and the SCD Probiotics supplementation group during intermittent fasting (n=7). The rats in the intermittent fasting groups were subjected to food restriction for 18 hours daily, with a 6-hour window (between 9 a.m. and 3 p.m.) for food intake. Water was available to them at all times. The animals were fed a typical rodent diet ad libitum [1] , and their body weight was recorded throughout the study. The probiotic supplement was administered orally at a dose of 3 mL (1 x 10^8^ CFU) per day [2] , using the product marketed by SCD Probiotics company (Essential Probiotics XI - 500 ml H.S. Code: 2206.00.7000), which contains 11 different probiotics, including *Bacillus subtilis, Bifidobacterium bifidum, Bifidobacterium longum, Lactobacillus acidophilus, Lactobacillus bulgaricus, Lactobacillus casei, Lactobacillus fermentum, Lactobacillus plantarum, Lactococcus lactis, Saccharomyces cerevisiae*, and *Streptococcus thermophilus* species. The 30-day treatment duration was chosen based on the standard protocols often used in pharmacological studies. Such a timeframe is commonly adopted to assess drug efficacy and observe potential long-term physiological changes in model organisms [3]. After the treatment, all animals were briefly anesthetized with ether treatment and sacrificed one day later. The liver tissues were extracted, immediately shocked on dry ice, and stored in a -80°C deep freezer until analysis [4].

**2.2 Analysis of samples by Attenuated Total Reflectance Fourier Transform Infrared (ATR-FTIR) spectroscopy**

For all animals, samples were taken from the liver and then directly placed on the Zn/Se crystal of the ATR unit from PerkinElmer for analysis. The samples were examined using an ATR-FTIR spectrometer from the same company, with a resolution of 4 cm^-1^ and a scan number 32. The data was collected using Spectrum One software from PerkinElmer, and the collected spectra were within the range of 4000-650 cm^-1^ wavelength [5–8].

**2.3 Prediction studies with different machine learning approaches based on big spectral data**

The study employed Linear Discriminant Analysis (LDA), a type of machine learning, to distinguish between the experimental groups. Spectral data were utilized in pattern recognition analysis, and to ensure the study was as independent as possible from the FTIR spectrometers, each sample spectrum was preprocessed using The Unscrambler® X 10.3 software from CAMO Software AS in Norway. Baseline offset transformation was applied in the 4000-650 cm^−1^ region, and the resulting spectra were first subjected to Principal Component Analysis (PCA), an unsupervised pattern processing technique. The spectra were also subjected to standard deviation normalization (mean centering normalization) and leverage or full-cross random validation. Then, the spectra were further analyzed in the lipid (3000-2700 cm^−1^), protein (1700-1500 cm^−1^), nucleic acid (1200-650 cm^−1^), and full (4000-650 cm^−1^) regions using the Singular Value Decomposition (SVD) algorithm.

LDA is a supervised classifier that transforms n-dimensional feature samples into an m-dimensional space. In contrast to PCA, which only transforms sample spectra, LDA incorporates class information from training samples for more accurate classification. This study used PCA data as LDA model inputs with the multivariate analysis (MVA) software, The Unscrambler® X 10.3 from CAMO Software AS in Norway. A data matrix was created, including the category variable column, and all spectra from different sample categories were used to generate a training set. The linear method, which utilizes the projections of the 9 PCA components, was employed for prediction, and prior probabilities were calculated from the training set. The results were presented as a discrimination plot, prediction, and confusion matrices [9].

Another popular machine-learning approach used in the study was the Support Vector Machine (SVM). The SVM classification method used The Unscrambler® X 10.3 multivariate analysis (MVA) software from CAMO Software AS in Norway. All spectra were pre-processed as described earlier, and different sample categories were used to generate a training set. Classification (nu-SVC) was chosen as the SVM type, with a linear method used as the Kernel type. The Nu value was set to 0.5, and all weights were set to 1.00. The training and cross-validation accuracies were calculated using the nine segments of cross-validation. Finally, the training dataset was applied to all sample datasets to obtain an SVM classification model [10].

**2.4 Quantification studies of FTIR spectral bands**

The OPUS 5.5 software from Bruker was used to perform spectral data analysis. To correct the baseline of each sample, the Rubberband correction method was used with 128 baseline points before the band quantification analyses. In a detailed band analysis, the bands with the highest absorbance values in different spectral regions were selected, and the beginning and ending frequencies of these bands were precisely determined. The integral areas of the determined frequency ranges were calculated using the OPUS 5.5 software to analyze the bands specific to various biomolecules. Additionally, a virtual line was drawn from the band baseline's midpoint to the band's peak, and the line length was measured using a virtual ruler. A horizontal line was drawn along the band from where 0.75 times the length coincides with the line, and bandwidth values were obtained [11].

**2.5 Histopathological analysis**

After tissue dissection, neutral-buffered formalin (10%) was used for the fixation of liver tissues for 48 h at room temperature, followed by preparation of paraffin sections (5 μm thickness) using a rotary microtome (Leica Biosystems, Germany). The obtained sections were stained using hematoxylin & eosin stain (H&E) (Hematoxylin: Cas N0: 517-28-2; Eosin: Cas N0: 17372-87-1, Merck, Germany) for a general architecture of the liver tissue. H&E slides were used to evaluate the rat liver's general tissue characterization. To investigate histological changes, an average of 10–15 areas was assessed by random sampling for each animal section in the groups [12]. Two researchers anonymously conducted microscopic analyses of the histopathological changes of the study.

**2.6 H&E staining**

For H&E staining, the following steps were performed respectively. Initially, all slides in each group were deparaffinized with xylene, dehydrated with ethanol, placed in a hematoxylin solution, then washed under running water. They were differentiated by acidic alcohol for a dip and then immediately rinsed with water, after which the slides were placed in eosin for 3 min. The next steps were dehydration in ascending ethanol and clearing in xylene. Subsequently, the slides were mounted by Entellan medium and visualized using an Olympus BX53 microscope with an Olympus DP74 camera attachment [13].

**2.7 Masson's trichrome (MT) staining**

MT staining was performed according to the instructions in the kit 04-010802 (Bio-Optica, Milan, Italy) to detect collagen accumulation of fibers in the rat liver. According to the commercial kit, the tissue sections were deparaffinized and rehydrated for MT staining. All procedures were carried out at room temperature.

**2.8 Quantification of histopathological parameters**

For histopathological H&E evaluations of lymphatic infiltration and microvesicular steatosis (micro lipid droplets) in the liver tissue, binarization in grayscale with a threshold set to the lowest level for detecting purple-stained and non-stained areas was used for quantifying area fractions (%) with Image J Fiji (National Institutes of Health, Bethesda, Maryland, USA). For MT staining, binarization in grayscale with a threshold set to the lowest level for detecting blue-stained areas was used for quantifying the MT-positive area with Image J Fiji. This quantification method was modified from [10]. For all animals in the same group, signal intensities from five images in each section were graphically shown. All microphotographs of the sections were analyzed by light microscopy (Olympus BX53, Japan) using a camera attachment (Olympus DP27, Japan) with imaging systems (Olympus cellSens Entry, Japan).

**2.9 Determination of Liver Biochemical Markers in Serum**

Blood taken from rats was centrifuged at 3500 rpm at + 4°C, and the serum was separated. Then, aspartate aminotransferase (AST), alanine aminotransferase (ALT), alkaline phosphatase (ALP), lactate dehydrogenase (LDH), and albumin levels were analyzed in a Hitachi C502 automated biochemical analyzer (Roche, Germany) with commercial kits (Roche Diagnostics). Enzyme activity of ALT, AST, ALP, LDH, and Albumin was expressed in international units per liter (IU/L) and g/L, respectively.

**2.10 Statistics**

Statistical evaluations and graph plots of the results were made using GraphPad Prism 9.01 (GraphPad, USA). The data were analyzed using One-way ANOVA and unpaired t-test, and the significance levels were stated as P <0.05 *, P ≤ 0.01 **, P ≤ 0.001 ***, and P ≤ 0.0001 ****. Results are presented as mean ± SEM (standard error of the mean).

**References**

[1] **Mattson MP, Longo VD, Harvie M**. Impact of intermittent fasting on health and disease processes. *Ageing Res Rev* 2017; 39; 46–58.

[2] **Zheng X, Wang S, Jia W**. Calorie restriction and its impact on gut microbial composition and global  metabolism. *Front Med* 2018; 12; 634–44.

[3] **Ceylani T, Teker HT, Keskin S, et al.** The rejuvenating influence of young plasma on aged intestine. *J Cell Mol Med* 2023; 27; 2804–16.

[4] **Teker HT, Ceylani T**. Intermittent fasting supports the balance of the gut microbiota composition. *Int Microbiol* 2022; doi: 10.1007/s10123-022-00272-7.

[5] **Gurbanov R, Karadağ H, Karaçam S, et al.** Tapioca Starch Modulates Cellular Events in Oral Probiotic Streptococcus salivarius Strains. *Probiotics Antimicrob Proteins* 2021; 13; 195–207.

[6] **Yonar D, Severcan M, Gurbanov R, et al.** Rapid diagnosis of malignant pleural mesothelioma and its discrimination from lung cancer and benign exudative effusions using blood serum. *Biochimica et Biophysica Acta (BBA) - Molecular Basis of Disease* 2022; 1868; 166473.

[7] **Karthikeyan S, Gurbanov R, Unal D**. Pb intoxicated biomolecular changes in Cladonia convoluta studied using 2DCOS infrared spectroscopy coupled with chemometric analysis. *Vib Spectrosc* 2022; 119; 103341.

[8] **Gurbanov R, Unal D**. The biomolecular alterations in cladonia convoluta in response to lead exposure. *Spectroscopy Letters* 2018; 51; 563–70.

[9] **Dogan A, Gurbanov R, Severcan M, et al.** CoronaVac (Sinovac) COVID-19 vaccine-induced molecular changes in healthy human  serum by infrared spectroscopy coupled with chemometrics. *Turk J Biol* 2021; 45; 549–58.

[10] **Ardahanlı İ, Özkan Hİ, Özel F, et al.** Infrared spectrochemical findings on intermittent fasting-associated gross  molecular modifications in rat myocardium. *Biophys Chem* 2022; 289; 106873.

[11] **Taner H, Taha T, Seda C, et al.** Age ‑ related differences in response to plasma exchange in male rat liver tissues : insights from histopathological and machine ‑ learning assisted spectrochemical analyses. *Biogerontology* 2023.

[12] **Erdogan K, Ceylani T, Teker HT, et al.** Young plasma transfer recovers decreased sperm counts and restores epigenetics in aged testis. *Exp Gerontol* 2023; 172; 112042.

[13] **Keskin S, Acikgoz E, Ertürk FY, et al.** Histopathological Changes in Liver and Heart Tissue Associated with Experimental  Ultraviolet Radiation A and B Exposure on Wistar Albino Rats. *Photochem Photobiol* 2023; 99; 132–6.

[14] **Adomshick V, Pu Y, Veiga-Lopez A**. Automated lipid droplet quantification system for phenotypic analysis of  adipocytes using CellProfiler. *Toxicol Mech Methods* 2020; 30; 378–87.
